# Effect of Hemodialysis on Retinal Thickness in Patients with Diabetic Retinopathy, with and without Macular Edema, Using Optical Coherence Tomography

**DOI:** 10.1155/2014/709862

**Published:** 2014-09-15

**Authors:** Nur Azem, Oriel Spierer, Meital Shaked, Meira Neudorfer

**Affiliations:** ^1^Departments of Ophthalmology, Tel Aviv Sourasky Medical Center, 6423906 Tel Aviv, Israel; ^2^The Sackler Faculty of Medicine, Tel Aviv University, 6997801 Tel Aviv, Israel; ^3^Departments of Nephrology, Tel Aviv Sourasky Medical Center, 6423906 Tel Aviv, Israel

## Abstract

*Background.* Effects of hemodialysis (HD) treatment on retinal thickness and macular edema are unclear. *Objective.* To evaluate changes in retinal thickness using optical coherence tomography (OCT) in end stage renal disease (ESRD) patients with diabetic retinopathy (DR), with and without diabetic macular edema (DME), undergoing HD. *Methods.* Nonrandomized prospective study. Forty eyes of DR patients with ESRD treated with HD were divided into two groups: patients with macular edema and patients without macular edema. Both eyes were analyzed. Patients underwent an ophthalmic examination including OCT measurements of retinal thickness, blood albumin and hemoglobin A1C levels, blood pressure, and body weight, 30 minutes before and after HD. *Results.* We found no significant effects of HD on retinal thickness among patients both with and without DME. The former showed a trend towards reduction in retinal thickness in foveal area following HD, while the latter showed an increase. There was no correlation between retinal thickness and mean blood pressure, weight, kinetic model value—*Kt*/*V*, glycemic hemoglobin, or albumin levels before and after HD. *Conclusions.* HD has no significant effect on retinal thickness among patients with or without DME. Further studies on larger cohorts and repeated OCT examinations are needed to confirm the preliminary findings in this study.

## 1. Introduction

End-stage renal disease (ESRD) patients undergoing hemodialysis have a higher incidence of ophthalmologic pathologies, including diabetic retinopathy (DR), exudative retinal detachment, and cataract [1, 2]. DR and diabetic macular edema (DME) are common microvascular complications of diabetes mellitus and are a major cause of decreasing visual acuity and blindness [3, 4]. Only a very small number of studies have examined the effects of hemodialysis on retinal thickness and DME, and those that did reported conflicting results. Some [5, 6] reported that hemodialysis is associated with resolution of hard exudates and a decrease in macular edema, while another stated that hemodialysis did not reduce diabetic macular leakage [7]. Those studies used fluorescein angiography, a technique that does not provide quantitative evaluation of macular edema and retinal thickness [8]. Optical coherence tomography (OCT), which both diagnoses and quantifies the degree of macular edema [9], is currently the gold standard for evaluating macular thickness [10]. There are currently only three published studies on the effect of hemodialysis on macular thickness in diabetic patients measured by OCT, with conflicting results. According to two of them [11, 12] hemodialysis decreased retinal thickness, while the third [13] demonstrated that foveal thickness did not change after one session of hemodialysis. We measured changes in retinal thickness in diabetic patients with and without macular edema by OCT before and after they underwent hemodialysis for ESRD. Having found no mention in the literature of gender effects of hemodialysis on retinal thickness, we evaluated this parameter as well.

## 2. Patients and Methods

Patients with ESRD due to diabetic mellitus who were undergoing hemodialysis in the Tel Aviv Sourasky Medical Center were recruited over a 2-year period. Those with DR, 18 years and older, regularly treated in the hemodialysis unit 3 times a week for 4 hours each time were invited to participate in the study. Exclusion criteria were retinal pathologies other than DR or opaque media (e.g., cataract), which precluded OCT images and inability to undergo the OCT examination due to mobility or cognitive difficulties. The study adhered to the tenets of the Declaration of Helsinki and was approved by the institutional Ethics Committee of our medical center. All participants signed an informed consent.

Each subject was administered a drop of tropicamide to dilate the pupils of the study eyes and then underwent an OCT examination 30 minutes before and 30 minutes after a hemodialysis session. OCT images were taken with the Stratus OCT direct cross-sectional imaging device-Zeiss Meditec (model 300, Dublin CA, USA 2006) using the macular thickness protocol. Six consecutive scans were performed for each eye. The OCT system then analyzed retinal thickness, creating a topographic map and graphs for quantitatively and qualitatively documenting any changes in retinal thickness and edema following hemodialysis. The quantitative topographic map was divided into nine parts; a central circle representing the fovea, and two rings, each divided into four quarters: the internal ring represented the thickness of the perifoveal area and the external ring the thickness of the peripheral macula.

Blood pressure and weight were measured before and after hemodialysis, and the delta of mean pressure (diastolic pressure + 1/3[systolic blood pressure − diastolic pressure]) was calculated. Glycemic hemoglobin and albumin levels were measured, as well as kinetic model—*Kt*/*V*, which is calculated monthly as an indicator of hemodialysis treatment efficacy (the desired value is 1.2–1.4) [14]. Clinical and laboratory data of male and female patients were compared.

## 3. Statistical Analysis

Data were recorded on Microsoft Excel spreadsheets. Right and left eyes were analyzed together. All analyses were two-tailed, and significance was set at *P* < .05. Statistical analyses were performed with SPSS software version 15.0 (SPSS, Inc., Chicago, IL). All data are expressed as mean ± SE. The level of change following hemodialysis was calculated as the difference between the two measurements. Retinal thickness, before and after hemodialysis, was compared by the paired *t*-test among all patients as a whole group and after dividing them to patients with and without macular edema. Percentage of change in retinal thickness was demonstrated by descriptive statistics. The correlation between change in retinal thickness and the HbA1C, albumin, *Kt*/*V*, delta of mean pressure, and delta of weight following hemodialysis were analyzed by Pearson correlation coefficient.

## 4. Results

The 23 subjects (40 eyes), 15 males and 8 females, ranged in age from 54 to 87 years. Six eyes that did not meet the inclusion criteria were excluded.

Table 1 summarizes the changes in retinal thickness among all patients before and after hemodialysis. There was a slight, not statistically significant decrease in retinal thickness among all the patients in all the examined areas, with the highest decrease in the foveal and perifoveal areas. The percent of change in retinal thickness before and after hemodialysis was very slight (Table 2).

Table 3 summarizes the clinical and laboratory parameters for Group A with macular edema (based on clinical impression and the presence of retinal cysts on OCT) and Group B without macular edema. The only significant difference between them was the mean blood pressure before and after hemodialysis. There were no significant group or gender differences in macular thickness changes at the central fovea, the inner macular ring, or the external macular ring. Group A displayed a trend towards reduction in macular thickness after hemodialysis in the foveal and perifoveal areas while Group B showed an increase of retinal thickness in the central area and a decrease in the perifoveal and peripheral macula, as shown in Table 4.

Comparisons of clinical and laboratory parameters between males and females disclosed two significant differences: diabetic disease duration was longer and the *Kt*/*V* value was higher in females. Overall, there was no significant difference between men and women in changes in retinal thickness, although men showed a tendency of decrease in retinal thickness in all areas and women showed a tendency of increase in all areas, without statistical significance.

There was no correlation between the changes in retinal thickness and mean blood pressure, weight, kinetic model value—*Kt*/*V*, glycemic hemoglobin, or albumin levels.

## 5. Discussion

We found no significant effects of hemodialysis on the retinal thickness of the eyes of diabetic subjects both with and without macular edema. The former showed only a trend towards reduction in retinal thickness in foveal area following hemodialysis, and the latter showed an increase in foveal area. There was no correlation between changes in retinal thickness and changes in albumin levels, hemoglobin A1C levels, weight, and mean blood pressure before and after hemodialysis.

The previously reported effects of hemodialysis treatment on retinal pathologies were based on fluorescein angiography [5, 7], which provides only qualitative information about the retina. The results of those reports were inconsistent. While Matsuo et al. [5] published a case report of two diabetic patients with ESRD who showed absorbance of residual lipids in the macula during a follow-up of 6 months after the initiation of hemodialysis, a prospective study by Takuyama et al. [7] found macular leakage unchanged at four weeks from the initiation of hemodialysis among such patients. Neither of these studies examined the effect of hemodialysis on retinal thickness.

Three recent studies employed OCT to evaluate the effect of hemodialysis on retinal thickness [11–13], some confirming our findings and others contradicting them. Pahor et al. [11] reported a significant reduction in retinal thickness among patients who required hemodialysis for different reasons, compared with healthy controls; the reduction was significant in all quadrants and was correlated with the age of the patients. This contradicts our finding of no change in retinal thickness following hemodialysis and might be due to differences in methodology: Pahor et al.'s subjects underwent a single OCT exam at time points independent of the hemodialysis session, while ours had OCT examinations 30 minutes before and 30 minutes after a hemodialysis treatment. We based the timing of the OCT in our study on previous reports [1, 15, 16] that hemodialysis patients who noted ocular side effects, such as blurred vision or eye pain, experienced them soon after the treatment, possibly due to the massive changes in osmolality caused by the hemodialysis. The different cohorts in our and Pahor et al.'s study could also account for the conflicting results: ours was all ESRD patients due to diabetes mellitus while theirs represented various etiologies.

Auyanet et al. [13] examined the change of average foveal thickness with OCT prior to and after hemodialysis among patients with type 2 diabetes mellitus, only one of whom had macular edema. Our study measured changes in retinal thickness at the central fovea as well as in the inner macular ring and the external macular ring in ESRD patients with type 2 diabetes mellitus. The foveal thickness in the study of Auyanet et al. [13] did not change after one session of hemodialysis in the 25 studied eyes apart from a slight (2%) and nonsignificant reduction. These results are compatible with ours despite the fact that their cohort included only one patient with macular edema. It also must be noted that Auganet and coworkers [13] did not mention the timing of the OCT exam.

Also using OCT, Theodossiadis et al. [12] found that hemodialysis treatment significantly decreased macular thickness among chronic renal failure diabetic patients with macular edema, with a less pronounced effect in the diabetic eyes without macular edema. Their patients, like ours, were examined 30 min before and 30 min after hemodialysis treatment. While these results are not compatible with ours, Theodossiadis et al.'s findings [12] of no correlation between changes in retinal thickness and changes in body weight and mean arterial blood pressure after hemodialysis are in agreement with our data. It is possible that, contrary to expectations, the systemic changes that occur during hemodialysis are not significant enough to change fluid balance and equilibrium [1, 15, 16].

During hemodialysis, osmotically active materials are eliminated by diffusion, resulting in a loss of body fluids and a decrease in blood osmolarity. This can affect the ocular parameters measured by OCT, including central foveal thickness, macular volume, retinal nerve fiber layer thickness, choroidal thickness, and IOP. We found two publications—Yang et al. [17] and Ulas et al. [18]—that evaluated choroidal changes pre- and posthemodialysis, mainly among nondiabetic patients. They demonstrated a decrease in choroidal thickness without any change in retinal thickness, in line with our findings.

We also noted that there was no difference between males and females in retinal thickness changes before and after hemodialysis, a parameter that has not been examined to date.

Our work has some limitations. The study population was relatively small and consisted solely of ESRD patients. In addition, we performed only two OCT examinations, 30 minutes prior to and 30 minutes after hemodialysis. Conducting more OCT exams at time points when the body had more time to achieve full equilibrium and fluid balance might have yielded different results. Furthermore, the OCT tests were performed without image registration, raising the possibility of different locations of the pre- and posthemodialysis measurements; this could explain why no significant changes were shown. The small number of eyes is also a limitation of the study, and further research on a larger sample is required to draw definitive conclusions about the differences in retinal thickness recorded before and after hemodialysis. Finally, the spectral domain OCT now available might have yielded results other than those we obtained with the time domain OCT.

While this pilot study turned up no significant changes in retinal thickness following hemodialysis in ESRD patients with DR, with and without DME, further research on a larger cohort with longer follow-up and diverse time frames is required to confirm the findings.

## Figures and Tables

**Table 1:** Difference between retinal thicknesses before versus after hemodialysis in diabetic retinopathy patients in different parts of the eye.

Parameter	Mean	Std. deviation	*P* value
Difference in retinal thickness in the foveal area	−3.32	28.37	0.541
Difference in retinal thickness in the perifoveal area	−4.45	21.07	0.274
Difference in retinal thickness in the peripheral macular area	−0.54	16.64	0.866

**Table 2:** Percent of change in retinal thicknesses before and after hemodialysis in diabetic retinopathy patients in different parts of the eye.

Parameter	Mean %	Std. deviation
Difference of retinal thickness in the foveal area	0.53	7.95
Difference of retinal thickness in the perifoveal area	1.4	7.13
Difference of retinal thickness in the peripheral macular area	0.92	5.95

**Table 3:** Clinical and laboratory data for diabetic retinopathy patients with (Group A) and without (Group B) macular edema undergoing hemodialysis.

Parameter	Group A (*n* = 19 eyes)	Group B (*n* = 21 eyes)	*P* value
Age (years)	73.36 ± 9.10	69.86 ± 11.49	0.380
Years of diabetic disease	12.50 ± 6.32	12.27 ± 6.47	0.930
Years of hemodialysis treatment	3.07 ± 1.94	3.38 ± 2.50	0.718
HbA1C (%)	6.17 ± 1.12	6.81 ± 1.45	0.212
Albumin (gram)	38.79 ± 3.33	37.71 ± 3.24	0.397
*Kt*/*V*—kinetic measure	1.45	1.38	0.399
Mean blood pressure difference (mm/Hg)—before and after hemodialysis	−4.92 ± 10.29	+5.25 ± 8.79	0.016
Weight difference (kg)—before and after hemodialysis	−1.92 ± 0.76	−1.73 ± 0.81	0.534

*Kt*/*V*—kinetic measure as indicator of hemodialysis efficacy.

**Table 4:** Difference between retinal thicknesses in different parts of the eye before and after hemodialysis in diabetic retinopathy patients with (Group A) and without (Group B) macular edema.

Parameter	Group A	Group B	*P* value
Difference of retinal thickness in the foveal area	−8.39 ± 38.69	1.75 ± 10.95	0.354
Difference of retinal thickness in the perifoveal area	−7.21 ± 25.42	−1.68 ± 16.11	0.497
Difference of retinal thickness in the peripheral macular area	2.21 ± 10.45	−3.29 ± 21.21	0.392
